# The Distinct Role of Small Heat Shock Protein 20 on HCV NS3 Expression in HEK-293T Cell Line

**Published:** 2018

**Authors:** Marzieh Basirnejad, Azam Bolhassani, Seyed Mehdi Sadat

**Affiliations:** 1. Department of Molecular and Cellular Sciences, Faculty of Advanced Sciences & Technology, Pharmaceutical Sciences Branch, Islamic Azad University, Tehran, Iran; 2. Department of Hepatitis and AIDS, Pasteur Institute of Iran, Tehran, Iran

**Keywords:** Hepatitis C virus, Small heat-shock proteins, Vaccines

## Abstract

**Background:**

Hepatitis C (HCV) is known as a serious blood-borne disease that infects millions of people globally. NS3 is a conserved non-structural sequence of hepatitis C virus which has a major role in activating specific CTL responses. As known, there is no effective vaccine against HCV infection, thus it is required to design a specific regimen of vaccination. Recently, the strong immunological properties of Heat shock proteins (Hsps) led to their use as immunomodulators and an antigen carrier for subunit vaccine candidates. In the current study, the role of Hsp20 was evaluated as a HCV NS3 gene carrier in mammalian cell line.

**Methods:**

At first, the recombinant plasmids of pEGFP-Hsp20, pEGFP-NS3, and pEGFP-Hsp20-NS3 were constructed and their accuracy was confirmed by digestion and sequencing. Then, all recombinant plasmids were transfected into HEK293T cells by Lipofectamine and TurboFect gene delivery systems. Finally, the expression of proteins was assessed by fluorescent microscopy, western blotting, and flow cytometry.

**Results:**

In western blotting, the 47, 59, and 79 *kDa* bands were detected for pEGFP-Hsp20, pEGFP-NS3, and pEGFP-Hsp20-NS3, respectively. The percentage of NS3-Hsp20-GFP protein expression was ∼67% by TurboFect and ∼50% by Lipofectamine indicating high potency of TurboFect delivery system. Furthermore, the expression of Hsp20 (∼83%) was higher than NS3 (∼58%) in the cells transfected by TurboFect using flow cytometry analysis. This result was confirmed in the expression of Hsp20-NS3 fusion (∼67%) in which Hsp20 increased the delivery of HCV NS3 *in vitro*. The same data were obtained by Lipofectamine transfection reagent.

**Conclusion:**

Briefly, our data confirmed the role of Hsp20 as a suitable antigen carrier for DNA vaccine design.

## Introduction

Hepatitis C virus (HCV) is a single-stranded enveloped RNA virus with nearly 9600 nucleotides in length. Based on the RNA genome of the virus, hepatitis C is categorized into six genotypes in the world that the most common type is genotype 1. Eleven proteins are encoded by HCV genome including structural and Non-Structural (NS) proteins. NS3 is a highly conserved, non-structural protein containing a serine protease domain in N-terminal and a helicase/NTPase domain in C-terminal of protein 1. NS3 was suggested to be the best vaccine candidate for hepatitis C due to the induction of strong T-cell immune responses against HCV NS3 related to clearance of infection. However, no effective HCV vaccine has been found because of the genetic variability of host defenses and the potential of the virus to escape the host immunity 2,3.

Among different vaccine strategies, DNA vaccines have attracted a specific interest due to easy production, heat resistant, and safety. A number of evidences showed that CpG motifs in plasmid vectors stimulate B-cell activity and subsequently humoral immune system 4. The aim of vaccination is to provide long-term protection against infections. Due to the low penetration of plasmid DNAs into the cells, development of an effective adjuvant is necessary for designing DNA vaccines 5. Therefore, researchers are studying for proper combination of antigens with effective adjuvants or carrier molecules in subunit vaccines 6.

Recently, Heat shock proteins (Hsps) were proposed to increase immune responses against infectious diseases 7. Hsps were classified into different families based on their molecular weight 8,9. Among heat shock proteins, small HSPs are highly conserved proteins among all species which have a conservative α-crystalline domain (∼90 amino acid residues). In addition, small HSPs have many functions such as protein folding, transportation, proteostasis, and immunity. Some small heat shock proteins are tissue specific in human such as HspB2, HspB3, α-crystalline (HspB4), HspB7, HspB9, and HspB10, while others are expressed in all human tissues including HspB1, αB-crystalline (Hsp-B5), HspB6 (20 *kDa*) and HspB8 10–17. HSPs are capable of delivering antigens to major histocompatibility complexes (MHC) for stimulation of adaptive immunity 6,7,18,19.

In this study, the plasmid DNAs encoding Hsp20, NS3, Hsp20-NS3 were generated and their expression was evaluated in mammalian cell line using a cationic polymer (TurboFect) and a cationic lipid (Lipofectamine). TurboFect transfection reagent is a cationic polymer in water which forms compact, stable and positively charged complexes with DNA facilitating gene delivery into eukaryotic cells (www.thermofisher.com). The data indicated that TurboFect transfection reagent was more effective than Lipofectamine for delivering the recombinant DNAs in HEK-293 T cells. Also, Hsp20 enhanced the transfection and expression of HCV NS3 *in vitro*. The obtained data would be a basic step for immunological studies in future.

## Materials and Methods

### Construction of the recombinant plasmids

**Figure 1.**
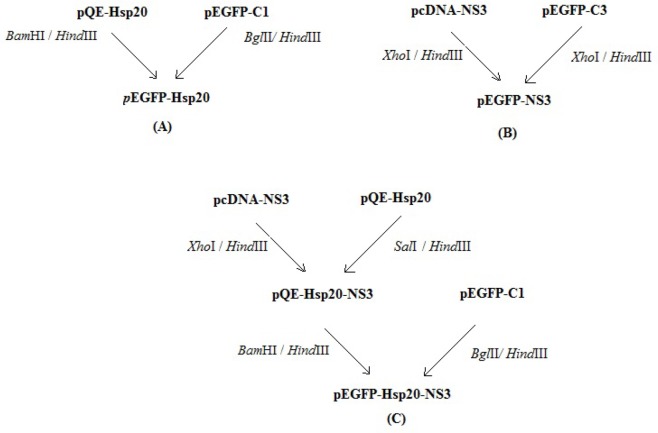
Schematic representation of cloning processes: Generation of pEGFP-Hsp20 (A); pEGFP-NS3 (B); pEGFP-Hsp20-NS3 (C) in bacterial system.

The full length of Hsp20 sequence was synthesized in pQE30 by Biomatik Company. For generation of pEGFP-Hsp20, the Hsp20 gene (Accession No: NM_001012401) was digested by *Bam*HI/*Hind*III and subcloned into *Bgl*II/*Hind*III sites of pEGFP-C1. The eukaryotic vector (pcDNA3.1) harboring the immunogenic and conserved region of HCV subtype 1a NS3 gene (1095-1379 aa, No: EU781798.1) was digested by *Xho*I/*Hind*III (Thermo scientific Fastdigest) and sub-cloned into pEGFP-C3 expression vector. To prepare the pEGFP-Hsp20-NS3, at first, the NS3 gene was ligated in *Sal*I/*Hind*III restriction sites of pQE-Hsp20 using T4 DNA ligase. Then, the fusion of Hsp20-NS3 was digested by *Bam*HI/*Hind*III and subcloned into *Bgl*II/*Hind*III cloning sites of pEGFP-C1. The *Escherichia coli (E. coli*) DH5α strain was transformed by all the recombinant vectors. After the extraction of plasmids from single colonies using DNA extraction kit (Qiagen), they were confirmed by digestion and sequencing. The recombinant pEGFP-NS3, pEGFP-Hsp20, and pEGFP-Hsp 20-NS3 plasmids were provided in large scale using DNA extraction kit (Qiagen, Germany) and quantified by NanoDrop spectrophotometry. The purity of plasmids was determined as OD_260_/OD_280_ ratio. This ratio was ∼1.85 for all plasmids indicating their purity. Figure 1 shows schematic representation of cloning process.

### Cell culture

Human embryonic kidney cells (HEK-293T) were cultured in Dulbecco’s Modified Eagle’s Medium (DMEM, Gibco) supplemented with 10% Fetal Bovine Serum (FBS, Gibco) at 37°*C* in presence of 5% CO_2_ atmosphere. After several passages using trypsin EDTA, the proliferated cells were counted by trypan blue 1X with hemocytometer and divided into 6-well plate.

### Transfection of plasmid DNAs into HEK-293T cells using Lipofectamine 2000

The day before transfection, the 5×10^5^ cells were counted and seeded into 6-well plates. The optimal cell confluency for effective transfection was considered 70–80%. For generation of Lipofectamine-plasmid DNA complex, 150 *μl* of serum-free medium was mixed with 10 *μl* of Lipofectamine (Invitrogen) and incubated for 5 *min* at room temperature. Then, 150 *μl* of incomplete DMEM was mixed with 4 *μg* of each plasmid (*i.e.* pEGFP-NS3, pEGFP-Hsp20, pEGFP-Hsp20-NS3, and pEGFP-C1 as a positive control), added to Lipofectamine solution, mixed gently, and incubated for 30 *min* at room temperature. After that, transfection complexes were added to each well and the medium was replaced after 6 *hr* of incubation at 37°*C* with the complete DMEM containing 10% FBS and 1/100 penicillin/streptomycin.

### Transfection of plasmid DNAs into HEK-293T cells using TurboFect

For generation of TurboFect-plasmid DNA complex, 7 *μl* of TurboFect (Thermo scientific) and 4 *μg* of plasmid were mixed and incubated for 15 *min* at room temperature. Finally, the complexes were added drop-wise to each well in serum-free medium. Six hours after the cell transfection, the medium was replaced with the complete DMEM medium. After 48 *hr*, transfection efficiency using TurboFect and also Lipofect-amine was evaluated by fluorescent microscopy, flow cytometry, and western blotting. The non-transfected cells and the cells transfected by pEGFP-C1 were used as negative and positive controls, respectively.

### Fluorescent microscopy and flow cytometry analysis

The quality and quantity of protein expression were monitored by GFP expression as a reporter gene using fluorescent microscopy (Envert Fluorescent Ceti, Korea) and Fluorescence-Activated Cell Sorting (FACS) caliber flow cytometer (Partec, Germany), respectively. For flow cytometry analysis, the cells were harvested by trypsin and the cell pellets were resuspended in 1 *ml* PBS (pH=7.4). Then, GFP expression in transfected cells was compared to non-transfected cells.

### Western blot analysis

For western blotting, the cells were harvested by trypsin, and the cell pellets were resuspended in PBS. Total cellular proteins were solved in 6X sample buffer containing Tris-HCl (0.5 *M*), glycerol, SDS, and 2-Mercaptoethanol (2%). The samples were separated on 15% acrylamide gel and transferred to nitrocellulose membrane. The membrane was incubated in blocking buffer (TBS 10X, 0.1% Tween 20, BSA, Merck) and washed with TBS10X and 0.1% Tween 20. Then, anti-GFP polyclonal antibody conjugated with horseradish peroxidase (1:10000 *v/v*) was used to detect the proteins of interest in the presence of DAB substrate (Ro-che Diagnostics-Germany).

### Statistical analysis

Statistical analysis (Student’s t-test) was performed by Prism 5.0 software (GraphPad, San Diego, California, USA) to analyze the percentage of NS3-GFP, Hsp20-GFP, and Hsp20-NS3-GFP expression using flow cytometry. The value of p<0.05 was considered statistically significant. Similar results were obtained in two independent experiments.

## Results

### DNA constructs expressing NS3, Hsp20, and Hsp20-NS3 genes

**Figure 2.**
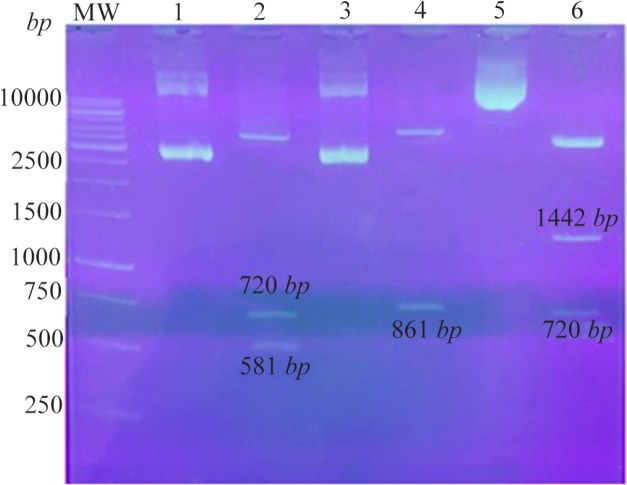
Confirmation of the recombinant plasmids using digestion: Lane 1: pEGFP-Hsp20, Lane 2: pEGFP-Hsp20 digested by *Nhe*I/*Hind*III (581 *bp*+720 *bp*), Lane 3: pEGFP-NS3, Lane 4: pEGFP-NS3 digested by *Xho*I/*Hind*III (861 *bp*), Lane 5: pEGFP-Hsp20-NS3, Lane 6: pEGFP-Hsp20-NS3 digested by *Nhe*I/*Hind*III (1442 *bp*+720 *bp*). MW is a molecular weight marker (1 *kb*, Fermentas).

The pEGFP plasmids are mammalian expression vectors containing GFP sequence and Kanamycin resistance marker which were selected in the current study. The recombinant plasmids were prepared by subcloning as mentioned in Methods section. The recombinant pEGFP-Hsp20 digested by restriction enzymes showed the clear bands of ∼720 and ∼581 *bp* related to GFP and Hsp20, respectively. The recombinant pEGFP-NS3 digested by enzymes indicated a ∼861 *bp* band related to NS3. In addition, the recombinant pEGFP-Hsp20-NS3 cut by *Nhe*I/*Hind*III showed the clear bands of 720 and 1442 *bp* related to GFP and Hsp20-NS3, respectively as shown in figure 2.

### Transient expression of proteins in HEK-293T cells

**Figure 3.**
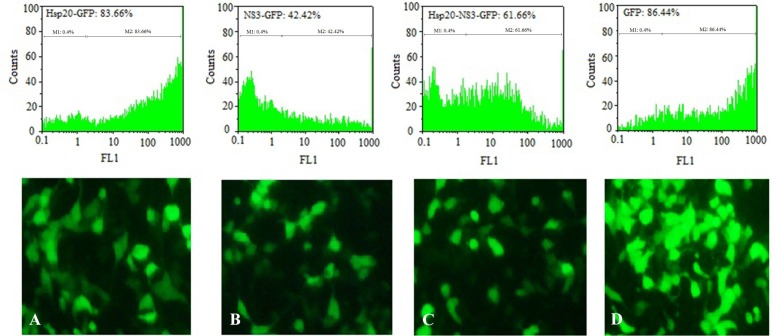
Transfection efficiency: Analysis of Hsp20-GFP (A), NS3-GFP (B), Hsp20-NS3-GFP (C), and GFP (D) protein expression in HEK-293T cells by TurboFect transfection reagent using fluorescent microscopy and flow cytometry. The pEGFP-C1 was used as a positive control (D). The non-transfected cell was considered as a negative control (M1).

**Figure 4.**
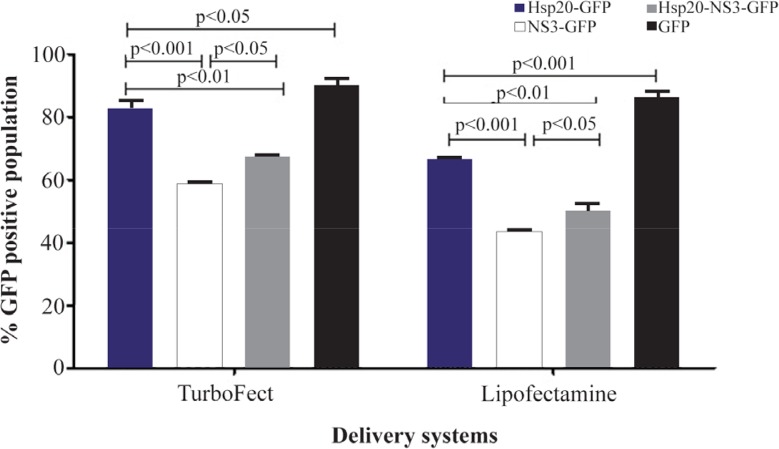
Comparison of TurboFect and Lipofectamine delivery in two independent experiments: The transfection efficiency of TurboFect was significantly higher than Lipofectamine.

The transfection efficiency of TurboFect and Lipo-fectamine reagents was confirmed by fluorescent microscopy and flow cytometry as shown in figure 3. Flow cytometry analysis indicated that the expression of pEGFP-Hsp20, pEGFP-NS3, and pEGFP-Hsp20-NS3 proteins using TurboFect transfection system was significantly higher than Lipofectamine (p<0.05). These results were obtained using the percentage of protein expression using GFP reporter marker. The per-centages of Hsp20-GFP, NS3-GFP, Hsp20-NS3-GFP, GFP expression were 83.10±2.24, 58.02±1.19, 67.23± 1.12, 90.15±1.99 for TurboFect system, and 66.01± 1.63, 43.31±0.85, 50.49±1.68, 86.45±1.75 for Lipofect-amine, respectively. Indeed, Hsp20 could enhance NS3 DNA delivery in the cells significantly more than NS3 DNA alone using both TurboFect and Lipofectamine transfection reagents (Figure 4, p<0.05).

### Western blotting

**Figure 5.**
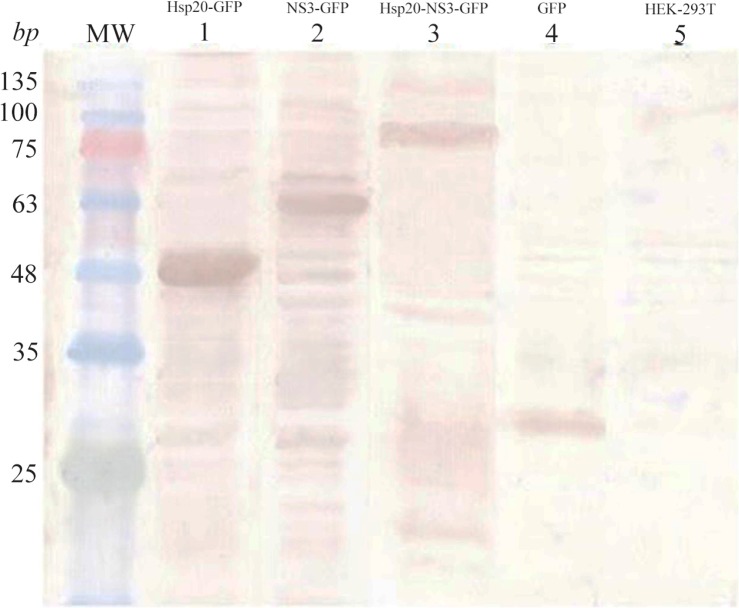
Identification of protein expression in HEK-293T cells 48 *hr* after transfection using western blot analysis: The expression of Hsp20-GFP (∼47 *kDa*, lane 1), NS3-GFP (∼59 *kDa*, lane 2), and Hsp20-NS3-GFP (∼79 *kDa*, lane 3) was detected by an anti-GFP antibody as compared to the non-transfected cells (lane 5). The GFP expression (∼27 *kDa*, lane 4) was applied as a positive control. MW is the molecular weight marker.

Western blot analysis indicated the successful expression of Hsp20-GFP, NS3-GFP, Hsp20-NS3-GFP, GFP proteins using anti-GFP antibody as shown in figure 5. The data indicated the clear bands of ∼47, ∼59, ∼79 and ∼27 *kDa* for Hsp20-GFP, NS3-GFP, Hsp20-NS3-GFP and GFP, respectively using DAB substrate.

## Discussion

The studies showed that HCV-specific CTL responses are very important for control of viral replication in chronically infected individuals 20. In 2001, Lazdina *et al* showed that NS3 elicits Th1 immune responses in a DNA vaccine significantly higher than that in a recombinant protein vaccine 21. In 1996, Missale *et al* examined the peripheral blood T cell proliferative responses against HCV core, E1, E2, NS3, NS4 and NS5 recombinant antigens. The results showed that 43% of T-cell responses were induced by NS3 antigen 22. The similar data were obtained by Tsai *et al* for a considerable CD4+ T-cell proliferation to NS3 in patients with acute hepatitis C 23.

In addition, the studies showed that NS3-specific immune responses are cross-reactive in various genotypes of HCV 24. Thus, NS3 was considered as a vaccine candidate. A major problem during development of DNA vaccines was their weak immunogenicity and the poor potency of such vaccines which may integrate into the host cell. For these reasons, there is a great necessity for the use of adjuvant to stimulate the T-cells 25.

In this study, HCV NS3 gene as an antigen candidate was effectively cloned in the eukaryotic expression vector alone or fused with Hsp20 as an effective carrier or adjuvant. The purity of plasmids was similar for using in transfection method. In general, the results showed that Hsp20 could increase the transfection efficiency of HCV NS3 in HEK-293T mammalian cells. There are some experiments for increasing the potency of HCV NS3 antigen. For instance, Naderi *et al* designated Interleukin-12 (IL-12) as an adjuvant for HCV NS3 DNA vaccine 26. Jiao *et al* showed that the cellular and humoral responses of the recombinant NS3 under the effect of CpG as an adjuvant was enhanced in animal model 27. Qazi *et al* used HSP70 as a suitable vaccine adjuvant. Based on this research, HSPs were selected as a carrier that conjugated to the malaria antigen EB200 and delivered both chimeric protein and DNA construct 28.

On the other hand, Barrios *et al* showed that mice immunized with peptides or oligosaccharides conjugated to the mycobacterial Hsp70 generated high titers of IgG antibodies in the absence of any adjuvant. Indeed, the use of Hsp as carrier in conjugated constructs for the induction of anti-peptide and anti-oligosaccharide antibodies could be of value in the design of novel vaccines 29. Ebrahimi and Tebianian also showed that the linkage of antigen with limited potency to an appropriate carrier such as C-terminal 28 *kDa* domain of mHS-P70 (HSP70_359–610_) containing an 18 *kDa* peptide binding region (aa 359–540) can increase its immunogenicity without any side effects 30. Moreover, Hsp20 contains α-crystalline domain known as a ligand for toll-like receptor 2/4 located on dendritic, macrophage, mast, monocyte, microglia, neutrophil, and T-cells 31.

Alvarez *et al* showed that the *Leishmania* Hsp20 as DNA vaccine is antigenic during natural infections. They indicated that 62% of the *Leishmania* infected animals, elicited considerable humoral responses against Hsp20 32. Ortiz *et al* reported that DNA fragment containing B and T cell epitopes of the N-terminal region of Hsp20 with other *Babesia bovis* antigens elicited high levels of specific IgG antibodies 33. Brown *et al* also reported that Hsp20 is a highly conserved protein between species and has B and T lymphocyte epitopes 34. Bepperling *et al* investigated bacterial small heat shock proteins such as Hsp17.7 and Hsp20.2 proteins. The results demonstrated that Hsp20.2 has strong chaperone activity as compared to Hsp17.7 35.

Regarding the roles of NS3 as an antigen candidate and Hsp20 as an antigen carrier and adjuvant, DNA constructs of pEGFP-NS3, pEGFP-Hsp20 and pEGFP-Hsp20-NS3 were prepared and their delivery was evaluated using TurboFect and Lipofectamine in mammalian cells. The researchers classified non-viral gene delivery systems in three groups: Naked DNA delivery, Lipid-based, and polymer-based delivery 36. Several studies used Lipofectamine and TurboFect as a vehicle to transport DNA constructs into the cells. In 1995, Lin *et al* transfected vHCV1027-1207 encoding the NS3 into BHK-21 cells with Lipofectamine 37. In 2003, He *et al* showed a suitable transfection of pRcHCNS3-5′ and pRcCMV expressing HCV NS3 into QSG7701 cells using lipofectamin 38. In 2004, Jiao *et al* studied the transfection of pSecTag2-HCV/NS3, pCI-HCV/NS3, and c3.1-HCV/NS3 into CHO-K1 cells by Lipofectamine. The NS3 expression was detected appropriately 39. In 2008, Lang *et al* observed that the expression of pCon-NS3/NS4A was confirmed through transient transfection of a Huh7.0 cell line with Lipofectamine 40.

In 2015, Behzadi *et al* described the expression of NS3 protein in Huh7 cells using Lipofectamine 41. In the same year, Bolhassani *et al* showed that TurboFect delivery system increased the efficiency of *in vitro* transfection of HCV core or coreE1E2 DNA 42. In the current study, the transfection efficiency of both delivery systems was compared (TurboFect and Lipofectamine) for delivering HCV NS3 DNA into HEK-293T cells. In addition, the ability of Hsp20 was evaluated to increase HCV NS3 expression in the cells. Our studies showed that the efficiency of TurboFect transfection reagent was significantly higher than Lipofectamine as a suitable tool for *in vitro* gene delivery. Moreover, Hsp20 could significantly enhance HCV NS3 DNA delivery and subsequently protein expression in HEK-293T cells using both delivery systems. These results confirm the role of Hsp20 as an antigen carrier.

## Conclusion

Generally, our data showed that the percentage of protein expression using TurboFect was higher than Lipofectamine reagent. In addition, the penetration of Hsp20 into the cells was significantly higher than NS3 using both transfection reagents.

## References

[1] Chevaliez S Pawlotsky JM, editors. HCV genome and life cycle. Norfolk (UK): Horizon Bioscience; 2006 451 p. (Tan SL, editor. Hepatitis C Viruses: Genomes and Molecular Biology). 21250393

[2] Larson AM Diagnosis and management of acute liver failure. Curr Opin Gastroenterol 2010;26(3):214–221. 20216412 10.1097/MOG.0b013e32833847c5

[3] Thimme R Oldach D Chang KM Steiger C Ray SC Chisari FV Determinants of viral clearance and persistence during acute hepatitis C virus infection. J Exp Med 2001;194(10):1395–1406. 11714747 10.1084/jem.194.10.1395 PMC2193681

[4] Sasaki S Takeshita F Xin KQ Ishii N Okuda K Adjuvant formulations and delivery systems for DNA vaccines. Methods 2003;31(3):243–254. 14511957 10.1016/s1046-2023(03)00140-3

[5] Petrovsky N Aguilar JC Vaccine adjuvants: current state and future trends. Immunol Cell Biol 2004;82(5): 488–496. 15479434 10.1111/j.0818-9641.2004.01272.x

[6] Qazi KR Qazi MR Julián E Singh M Abedi-Valugerdi M Fernández C Exposure to mycobacteria primes the immune system for evolutionarily diverse heat shock proteins. Infect Immun 2005;73(11):7687–7696. 16239573 10.1128/IAI.73.11.7687-7696.2005 PMC1273840

[7] Suzue K Young RA Heat shock proteins as immunological carriers and vaccines. EXS 1996;77:451–465. 8856990 10.1007/978-3-0348-9088-5_30

[8] van Noort JM Bsibsi M Nacken P Gerritsen WH Amor S The link between small heat shock proteins and the immune system. Int J Biochem Cell Biol 2012;44(10): 1670–1679. 22233974 10.1016/j.biocel.2011.12.010

[9] Kampinga HH Garrido C HSPBs: small proteins with big implications in human disease. Int J Biochem Cell Biol 2012;44(10):1706–1710. 22721753 10.1016/j.biocel.2012.06.005

[10] Basha E O’Neill H Vierling E Small heat shock proteins and α-crystallins: dynamic proteins with flexible functions. Trends Biochem Sci 2012;37(3):106–117. 22177323 10.1016/j.tibs.2011.11.005 PMC3460807

[11] Bakthisaran R Tangirala R Rao ChM Small heat shock proteins: Role in cellular functions and pathology. Biochim Biophys Acta 2015;1854(4):291–319. 25556000 10.1016/j.bbapap.2014.12.019

[12] Lindquist S The heat-shock response. Annu Rev Biochem 1986;55(1):1151–1191. 2427013 10.1146/annurev.bi.55.070186.005443

[13] Taylor RP Benjamin IJ Small heat shock proteins: a new classification scheme in mammals. J Mol Cell Cardiol 2005;38(3):433–444. 15733903 10.1016/j.yjmcc.2004.12.014

[14] Kim YE Hipp MS Bracher A Hayer-Hartl M Hartl FU Molecular chaperone functions in protein folding and proteostasis. Annu Rev Biochem 2013;82:323–355. 23746257 10.1146/annurev-biochem-060208-092442

[15] Velichko AK Markova EN Petrova NV Razin SV Kantidze OL Mechanisms of heat shock response in mammals. Cell Mol Life Sci 2013;70(22):4229–4241. 23633190 10.1007/s00018-013-1348-7 PMC11113869

[16] Kappé G Franck E Verschuure P Boelens WC Leunissen JA de Jong WW The human genome encodes 10 alpha-crystallin-related small heat shock proteins: HspB1-10. Cell Stress Chaperones 2003;8(1):53–61. 12820654 10.1379/1466-1268(2003)8<53:thgecs>2.0.co;2 PMC514853

[17] Sun Y MacRae TH Small heat shock proteins: molecular structure and chaperone function. Cell Mol Life Sci 2005;62(21):2460–2476. 16143830 10.1007/s00018-005-5190-4 PMC11138385

[18] Bolhassani A Rafati S Heat-shock proteins as powerful weapons in vaccine development. Expert Rev Vaccines 2008;7(8):1185–1199. 18844593 10.1586/14760584.7.8.1185

[19] Newport GR Heat shock proteins as vaccine candidates. Semin Immunol 1991;3(1):17–24. 1893121

[20] Diepolder HM Zachoval R Hoffmann RM Wierenga EA Santantonio T Jung MC Possible mechanism involving T-lymphocyte response to non-structural protein 3 in viral clearance in acute hepatitis C virus infection. Lancet 1995;346(8981):1006. 7475549 10.1016/s0140-6736(95)91691-1

[21] Lazdina U Hultgren C Frelin L Chen M Lodin K Weiland O Humoral and CD4(+) T helper (Th) cell responses to the hepatitis C virus non-structural 3 (NS3) protein: NS3 primes Th1-like responses more effectively as a DNA-based immunogen than as a recombinant protein. J Gen Virol 2001;82(6):1299–1308. 11369873 10.1099/0022-1317-82-6-1299

[22] Missale G Bertoni R Lamonaca V Valli A Massari M Mori C Different clinical behaviors of acute hepatitis C virus infection are associated with different vigor of the anti-viral cell-mediated immune response. J Clin Invest 1996;98(3):706–714. 8698862 10.1172/JCI118842 PMC507480

[23] Tsai S Liaw Y Chen MH Huang CY Kuo GC Detection of type 2-like T-helper cells in hepatitis C virus infection: Implications for hepatitis C virus chronicity. Hepatology 1997;25(2):449–458. 9021963 10.1002/hep.510250233

[24] Sällberg M Zhang ZX Chen M Jin L Birkett A Peterson DL Immunogenicity and antigenicity of the ATPase/helicase domain of the hepatitis C virus non-structural 3 protein. J Gen Virol 1996;77(11):2721–2728. 8922465 10.1099/0022-1317-77-11-2721

[25] Gurunathan S Klinman DM Seder RA DNA vaccines: immunology, application, and optimization. Annu Rev Immunol 2000;18(1):927–974. 10837079 10.1146/annurev.immunol.18.1.927

[26] Naderi M Saeedi A Moradi A Kleshadi M Zolfaghari MR Gorji A Interleukin-12 as a genetic adjuvant enhances hepatitis C virus NS3 DNA vaccine immunogenicity. Virol Sin 2013;28(3):167–173. 23709057 10.1007/s12250-013-3291-z PMC8208352

[27] Jiao X Wang RY Qiu Q Alter HJ Shih JW Enhanced hepatitis C virus NS3 specific Th1 immune responses induced by co-delivery of protein antigen and CpG with cationic liposomes. J Gen Virol 2004;85(Pt 6):1545–1553. 15166438 10.1099/vir.0.79896-0

[28] Qazi KR Wikman M Vasconcelos NM Berzins K Stahl S Fernández C Enhancement of DNA vaccine potency by linkage of Plasmodium falciparum malarial antigen gene fused with a fragment of HSP70 gene. Vaccine 2005;23(9):1114–1125. 15629354 10.1016/j.vaccine.2004.08.033

[29] Barrios C Lussow AR Van Embden J Van Der Zee R Rappuoli R Costantino P Mycobacterial heat-shock proteins as carrier molecules. II: The use of the 70-kDa mycobacterial heat-shock protein as carrier for conjugated vaccinescan circumvent the need for adju-vants and Bacillus Calmette Guérin priming. Europ J Immunol 1992;22(6):1365–1372. 10.1002/eji.1830220606 1601031

[30] Ebrahimi SM Tebianian M Role of mycobacterial heat shock protein 70 (mHSP70) as genetic vaccine adjuvants. World Appl Sci J 2011;14(10):1569–1575.

[31] McNulty S Colaco CA Blandford LE Bailey CR Baschieri S Todryk S Heat-shock proteins as dendritic cell-targeting vaccines-getting warmer. Immunology 2013;139(4):407–415. 23551234 10.1111/imm.12104 PMC3719058

[32] Montalvo-Alvarez AM Folgueira C Carrión J Monzote-Fidalgo L Cañavate C Requena JM The Leishmania HSP20 is antigenic during natural infections, but, as DNA vaccine, it does not protect BALB/c mice against experimental L. amazonensis infection. J Biomed Biotechnol 2008;2008:695432. 18401455 10.1155/2008/695432 PMC2288687

[33] Jaramillo Ortiz JM Del Médico Zajac MP Zanetti FA Molinari MP Gravisaco MJ Calamante G Vaccine strategies against Babesia bovis based on prime-boost immunizations in mice with modified vaccinia Ankara vector and recombinant proteins. Vaccine 2014;32(36): 4625–4632. 24968152 10.1016/j.vaccine.2014.06.075

[34] Brown WC Ruef BJ Norimine J Kegerreis KA Suarez CE Conley PG A novel 20-kilodalton protein conserved in Babesia bovis and B. bigemina stimulates memory CD4(+) T lymphocyte responses in B. bovis-immune cattle. Mol Biochem Parasitol 2001;118(1):97–109. 11704278 10.1016/s0166-6851(01)00375-9

[35] Bepperling A Alte F Kriehuber T Braun N Weinkauf S Groll M Alternative bacterial two-component small heat shock protein systems. Proc Natl Acad Sci USA 2012;109(50):20407–20412. 23184973 10.1073/pnas.1209565109 PMC3528540

[36] Park TG Jeong JH Kim SW Current status of polymeric gene delivery systems. Adv Drug Deliv Rev 2006; 58(4):467–486. 16781003 10.1016/j.addr.2006.03.007

[37] Lin C Thomson JA Rice CM A central region in the hepatitis C virus NS4A protein allows formation of an active NS3-NS4A serine proteinase complex in vivo and in vitro. J Virol 1995;69(7):4373–4380. 7769699 10.1128/jvi.69.7.4373-4380.1995 PMC189178

[38] He QQ Cheng RX Sun Y Feng DY Chen ZC Zheng H Hepatocyte transformation and tumor development induced by hepatitis C virus NS3 c-terminal deleted protein. World J Gastroenterol 2003;9(3):474–478. 12632500 10.3748/wjg.v9.i3.474 PMC4621564

[39] Jiao X Wang RY Feng Z Hu G Alter HJ W-K Shih J DNA immunization encoding the secreted nonstructural protein 3 (NS3) of hepatitis C virus and enhancing the Th1 type immune response. J Viral Hepat 2004;11(1):18–26. 14738554 10.1046/j.1352-0504.2003.00464.x

[40] Lang KA Yan J Draghia-Akli R Khan A Weiner DB Strong HCV NS3-and NS4A-specific cellular immune responses induced in mice and Rhesus macaques by a novel HCV genotype 1a/1b consensus DNA vaccine. Vaccine 2008;26(49):6225–6231. 18692108 10.1016/j.vaccine.2008.07.052 PMC4477808

[41] Behzadi MA Alborzi A Pouladfar G Dianatpour M Ziyaeyan M Expression of NS3/NS4A proteins of Hepatitis C virus in Huh7 cells following engineering its eukaryotic expression vector. Jundishapur J Microbiol 2015;8(11):e27355. 26862385 10.5812/jjm.27355 PMC4741058

[42] Mehrlatifan S Mirnurollahi SM Motevalli F Rahimi P Soleymani S Bolhassani A The structural HCV genes delivered by MPG cell penetrating peptide are directed to enhance immune responses in mice model. Drug Deliv 2016;23(8):2852–2859. 26559939 10.3109/10717544.2015.1108375

